# A Contribution to the Physiology of the Plastic Art

**Published:** 1880-02

**Authors:** James I. Tucker

**Affiliations:** Chicago


### ARTICLE XIII.

### A CONTRIBUTION TO THE PHYSIOLOGY OF THE PLASTIC ART.

*Messrs. Editors* :—It is plainly the duty of each, the busy practitioner and the still busier student, not to wait for time and opportunity to work out long and difficult scientific problems, but, being constantly in the presence of important facts, to contribute anything, however apparently trivial, coming within the scope of observation and experience, to the general store of medical knowledge. The following is not unimportant in a purely medical point of view and it is certainly a medical novelty of considerable physiological significance.

My office is directly opposite the unpretentious and busy studio of the eminent sculptor, Mr. Leonard W. Volk. In the intervals of practice it has been my privilege, and I have improved the occasions, to watch the process of modelling in clay, a process which is familiar to every school-boy in Rome, but rarely witnessed by the citizen of Chicago, and scarcely less rarely in other parts of America. The sculptor has for more than a year, been engaged upon five colossal statues; the first being that of ex-Senator Douglas and the other four being symbolical figures representing "Illinois," "History," "Justice," and "Eloquence," which are to surround the base of the monument. What struck me forcibly and led me to write this brief communication, was the number and variety of necessary manipulations, the patting, coaxing, spreading, the rubbing out and filling up and sticking on of the plastic clay, before the beautiful statue was ready to be immortalized in bronze. These manipulations and movements were so frequent and numerous that I was curious to follow and even to count them. I did this with the following striking results: Mr. Volk is actively at work in his studio every day from six to twelve hours. He naturally works more hours during the long days of summer. He works incessantly at least eight hours every day. This is a fair average. It took him one hundred and twenty days to create the statue of Douglas and he occupied ninety days each in creating the other four. Multiplying the number of days by eight, the number of hours he works each day, and we get four thousand, eight hundred and forty hours, which, reduced to minutes, yields two hundred and thirty thousand and four hundred. The number of strokes was constantly from one hundred and twenty to one hundred and sixty a minute. We will use the minimum figure to allow for rest and interruptions. Multiplying by this we get the following, viz., *twenty-seven million, six hundred and forty-eight thousand strokes!* Now, most of these manipulations are performed by the index-finger and thumb of the right hand, and when modelling-sticks are used there is still no rest for these two members. Mr. Volk fortunately has become ambi-dextrous since he received an injury to the right thumb and thus he can throw a part of the labor on the thumb and finger of the left hand. One would naturally suppose that the muscles so incessantly engaged in these delicate manipulations would become the subject of neuroses like the kinesiopareses, paralyses and cramps of writers, pianists, and violinists, but notwithstanding the artist, unlike them, works in cold and wet material, he assures me that he has never suffered from any inconvenience beyond a myalgia of the muscles which are exercised, fatigued and chilled, and a soreness due to the wearing down close of the finger nails, especially when the clay is gritty. One would naturally expect more marked results from *thirty millions of movements in the course of a single year mainly by one finger and one thumb!* In the calculation I have made above I have not taken into account at all the work required to reproduce the clay model in plaster, a part of the art of sculpture which only American sculptors have to do themselves. It is a purely mechanical operation, of course, but it is very laborious, and is always done by common skilled artizans in Italy.

DR. JAMES I. TUCKER.

CHICAGO.

